# Giant cutaneous metastasis from hepatocellular carcinoma

**DOI:** 10.1002/jgh3.12743

**Published:** 2022-04-23

**Authors:** Asahiro Morishita, Joji Tani, Kyoko Oura, Tomoko Tadokoro, Koji Fujita, Tsutomu Masaki

**Affiliations:** ^1^ Department of Gastroenterology and Neurology Kagawa University Faculty of Medicine Kita‐gun Kagawa Japan

**Keywords:** cutaneous metastasis, facial verruca, hepatocellular carcinoma

## Abstract

Cutaneous metastases from hepatocellular carcinoma (HCC) are rare and mostly occur on the face, scalp, chest, and shoulders, often with rapid growth. We report the case of an 86‐year‐old man who presented to our hospital with a mass, 5 cm in diameter, on the left side of his nose. His past history included a partial hepatectomy for HCC, 3 years previously, and a subsequent diagnosis of lung metastases. Skin and gingival biopsies confirmed the diagnosis of metastatic HCC and he was treated with immunotherapy followed by transarterial embolization (TAE). The latter resulted in a significant reduction in the cutaneous tumour mass.

## Graphical abstract

We report a rare case of giant cutaneous metastasis on the face from hepatocellular carcinoma. This skin nodule was rapidly developed and transarterial embolization was effective for inhibiting tumour development.

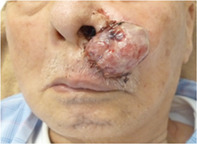

## Introduction

Hepatocellular carcinoma (HCC) with cutaneous metastases are extremely rare, and these skin lesions rapidly develop. The preferred site of skin metastasis from HCC is mostly the face, scalp, chest, and shoulders, and these lesions appear singly or in multiples as firm, painless, nonulcerative, reddish nodules.^1^, ^2^

## Case Report

**Figure 1:**
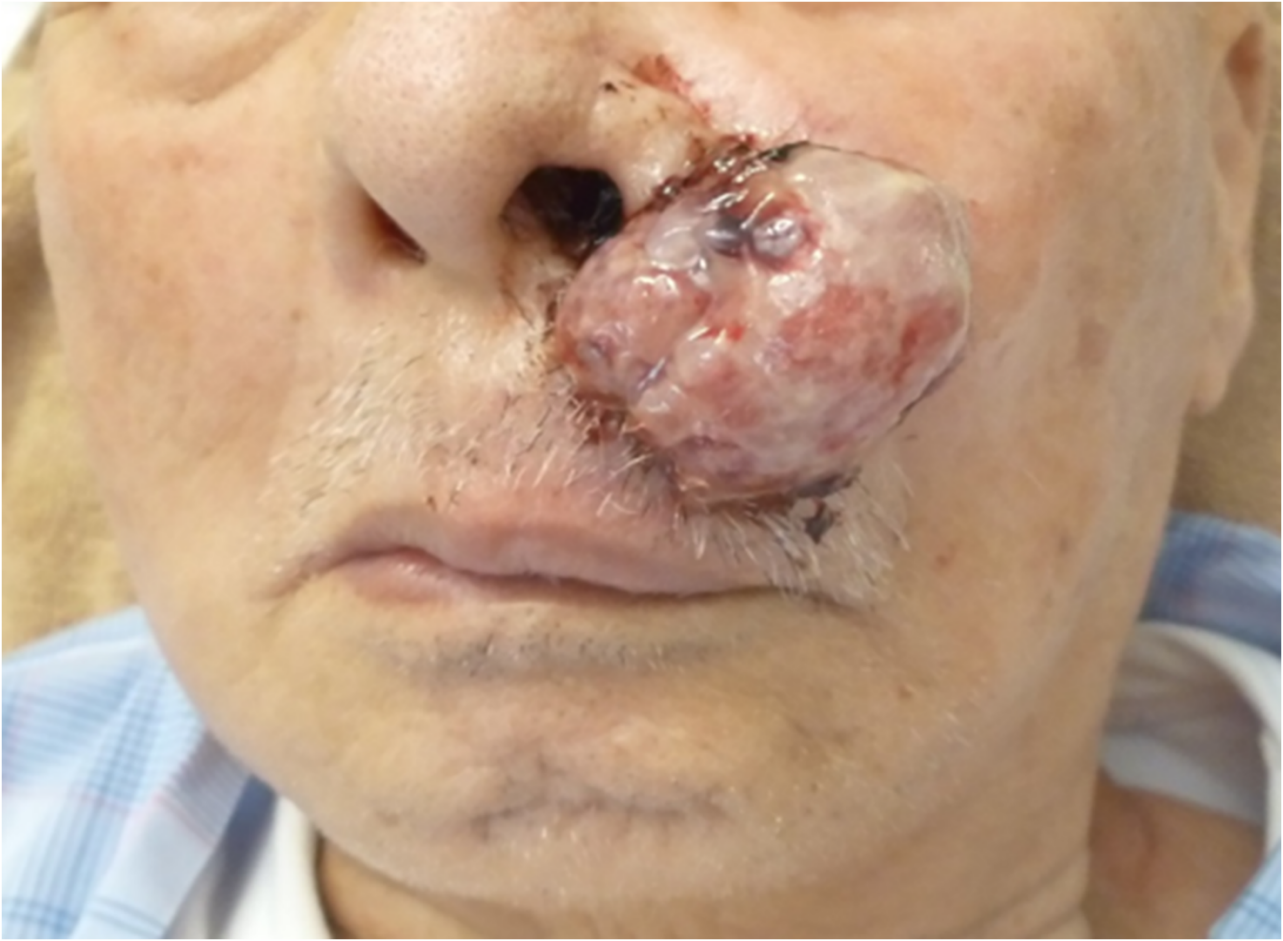
A verruca on the left nose wing. A large, pedunculated verruca extending from the left upper gingiva.

An 86‐year‐old man presented to our hospital with a giant verruca on his left nose wing (Fig. 1) in July 2020. He had a medical history of partial hepatectomy due to a rupture of moderately differentiated HCC 3 years earlier. The margins of the tumor were clear after surgery. Thirteen months after operation, lung metastases were detected by enhanced computed tomography (CT) on follow‐up. He has been administrated several multi‐molecular target agents (MTAs). The effects of MTAs indicated stable disease, as evaluated by the modified response evaluation criteria in solid tumors (mRECIST).

**Figure 2:**
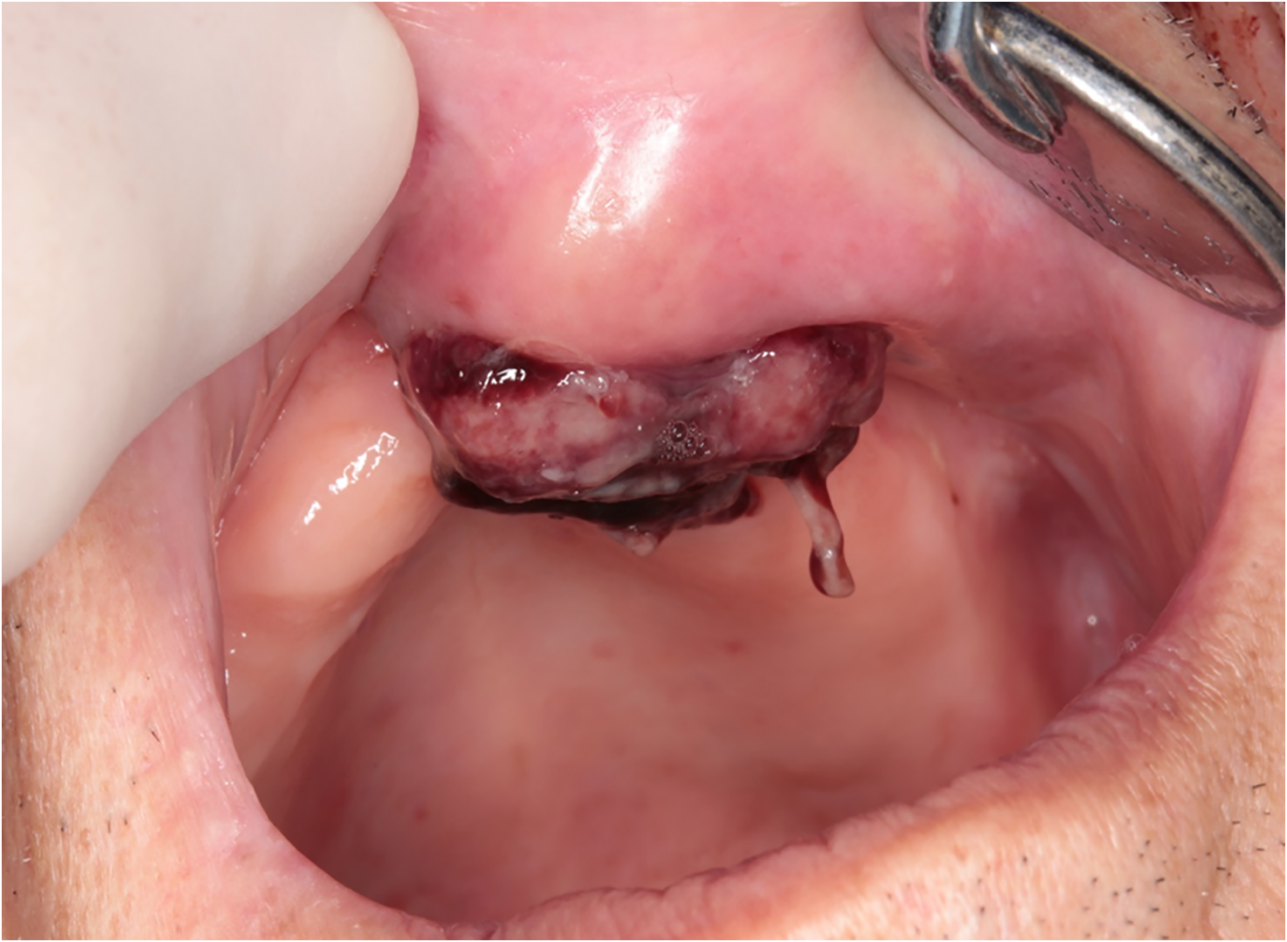
A large, pedunculated verruca extending from the left upper gingiva.

**Figure 3:**
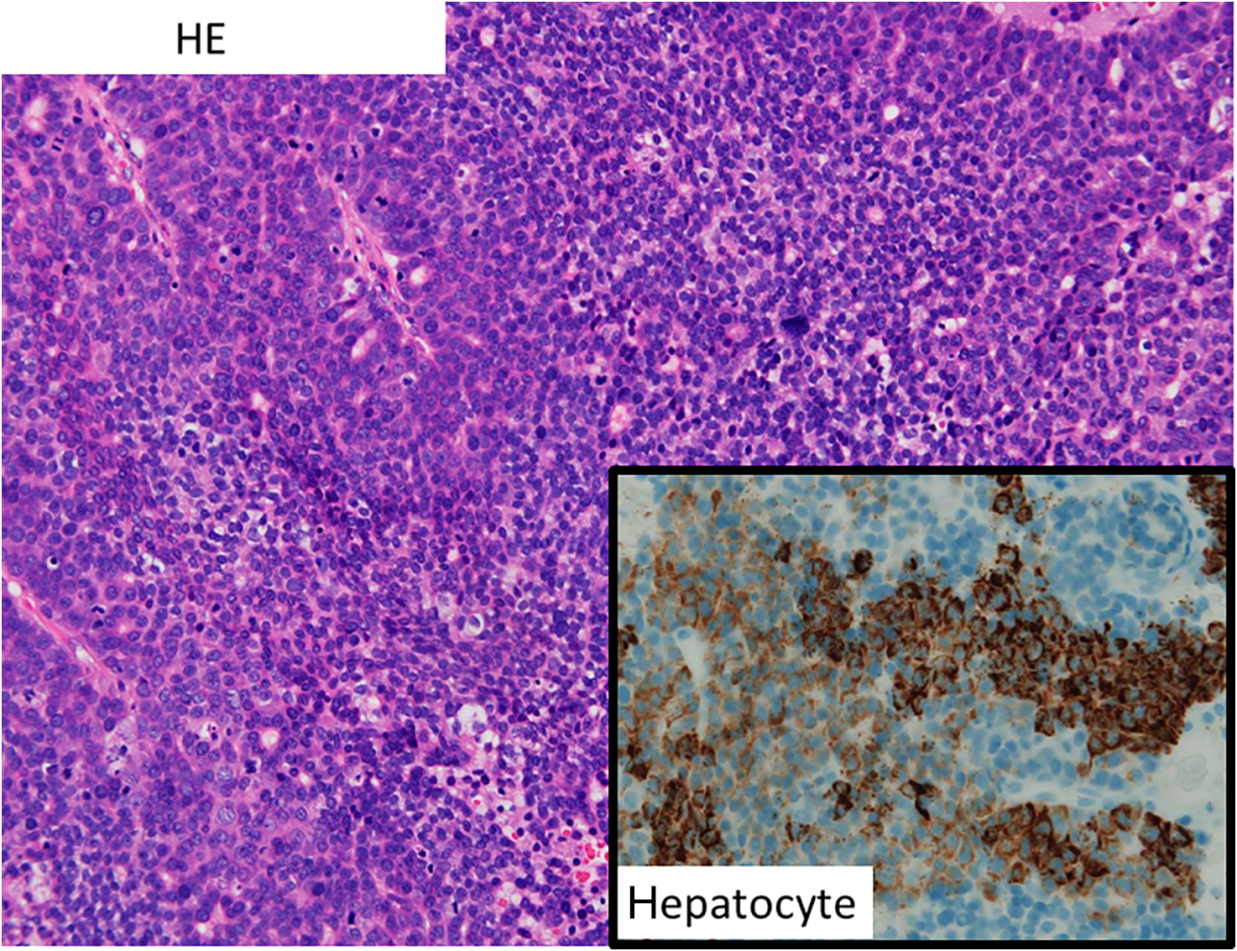
Histopathology showing a poorly differentiated hepatocellular carcinoma with hepatocyte (+).

He first noticed the mass after bleeding occurred while he was brushing his teeth. Two months later, a wart was detected on his left nose wing, which gradually increased in size (Fig. 1). On examination, a large, non‐tender, pedunculated mass was seen extending from left upper gingiva (Fig. 2). The surface was friable and bled easily. This tumor had penetrated the gums. An excisional biopsy was performed, and histopathological analysis showed a poorly differentiated HCC with hepatocyte (+) (Fig. 3). A staging CT confirmed multiple bilateral pulmonary metastases but no liver metastasis. He was offered immunotherapy for metastatic HCC involving the skin and lungs with a combination of atezolizumab and bevacizumab. He was administered 10 cycles of atezolizumab and bevacizumab (1200 mg of atezolizumab plus 15 mg per kilogram of body weight of bevacizumab intravenously every 3 weeks). Although the lung nodules remained stable on a repeat CT of the thorax, the cutaneous metastasis increased in size. Therefore, transarterial embolization (TAE) was performed for the cutaneous giant HCC. This resulted in shrinkage of the cutaneous lesion. Nearly 30% of the volume of the cutaneous metastatic tumor was reduced without ulceration or necrosis on its surface. The patient was discharged 7 days after TAE. Cutaneous metastatic tumor on the face was controlled, and the patient survived for more than 10 months after TAE.

## Discussion

Cutaneous metastases of HCC are extremely rare and only a few case reports exist. Of these reports, several describe cutaneous metastases due to direct engraftment of HCC by biopsy or procedures including percutaneous ethanol injection therapy.^3^ Such direct deposition is of course different in etiology from the distant metastasis we report here. Previous reports have shown that skin lesions present as single or multiple firm, painless, nonulcerative, reddish nodules that are approximately 1–2.5 cm in diameter. These skin lesions appear on the face, scalp, chest, and shoulders and show rapid growth.^4^, ^5^ In this study, a giant mass of more than 5 cm was seen extending from the left upper gingiva in 2 months. Therefore, the possibility of skin metastasis should be considered in HCC patients who present with skin nodules, and the diagnosis should be confirmed by biopsy immediately. The face and scalp are commonly involved, which is thought to result from arteriovenous shunts that facilitate the tumor to spread up the inferior vena cava to the head and neck. Because other atypical sites of HCC metastasis have been reported, including the thyroid gland, mediastinum, or ovaries, it is important to remember that metastatic recurrence of HCC can present unconventionally several years after an initial HCC treatment, and close attention should be paid to new complaints such as skin lesions that might easily be missed.

## References

[1] Kubota Y , Koga T , Nakayama J . Cutaneous metastasis from hepatocellular carcinoma resembling pyogenic granuloma. Clin. Exp. Dermatol. 1999; 24: 78–80. 10233658 10.1046/j.1365-2230.1999.00423.x

[2] Reingold IM , Smith BR . Cutaneous metastases from hepatomas. Arch. Dermatol. 1978; 114: 1045–6. 210712

[3] Nagaoka Y , Nakayama R , Iwata M . Cutaneous seeding following percutaneous ethanol injection therapy for hepatocellular carcinoma. Intern. Med. 2004; 43: 268–9. 15098615 10.2169/internalmedicine.43.268

[4] Asselah T , Condat B , Cazals‐Hatem D *et al*. Ectopic hepatocellular carcinoma arising in the left chest wall: a long‐term follow‐up. Eur. J. Gastroenterol. Hepatol. 2001; 13: 873–5. 11474320 10.1097/00042737-200107000-00018

[5] Wood AJ , Lappinga PJ , Ahmed I . Hepatocellular carcinoma metastatic to skin: diagnostic utility of antihuman hepatocyte antibody in combination with albumin in situ hybridization. J. Cutan. Pathol. 2009; 36: 262–6. 18727662 10.1111/j.1600-0560.2008.01029.x

